# Dr. Adams on M. Ramond's Account of Gôitre in Our Last Number (See P. 209); with the Editor's Answer

**Published:** 1815-04

**Authors:** Joseph Adams

**Affiliations:** Hatton-Garden.


### For the Medical and Physical Journal.

### Dr. Adams on *M. Ramond's Account of Gôitre in our last Number* (see p. 209); *with the Editor's Answer.*

YOUR last Number (p. 209) contains a valuable history of that unhappy and degraded part of our species usually known under the names of *Cretins* or *Gôitres*. At the commencement and close of your extracts, a few words are added by the Editor, referring to some writer whose name you have not thought proper to insert. Perhaps, it may be only from the general feelings of an author, that I sometimes fancy you may have referred to my publication on "Hereditary Diseases." If so, I will thank you to copy the passage to which you allude; after which, I trust yourselves and your readers will be convinced, that my opinion of the origin of this race is very different from M. Ramond's. I am, however, ready to allow, that the perusal of M. Ramond's history might have led to my inferences, and most gladly should I have availed myself of his account had it come to my knowledge before my own little work was published. To convince you how useful such a paper would have been to me, I shall, if you will permit me, in your next Number, offer some remarks upon it, and the confirmation which it seems to afford "that the same causes [the breeding *in and in*] operating in man, as Sir John Seabright informs us—and as Colonel Humfries proved experimentally in other animals, might have produced many endemic peculiarities in certain sequestered districts, which have hitherto been imputed to the water and other localities."*

*Hatton-Garden.* JOSEPH ADAMS, M.D.

**Note from the Editors.**—We are ready to admit, that Dr. Adams's work was the one to which we alluded.—We need not say how willingly we shall receive his communication, which we hope will arrive in time to appear in our next Number. The following is the note subjoined to that part of the text, which is inserted in the letter above, and which, much more than the text, induced us to add the remark at the end of our quotation from M. Ramond.

"NOTE 19, p. 34.—'*Many endemic peculiarities found in certain sequestered districts, which have been imputed to the water and other localities.*'

"The prevalence of certain diseases in particular districts, has always been an object of inquiry. Hippocrates ascribes many of them to the water and air of the places. This opinion has pre-

* Hereditary Diseases, p. 33 and 34.

vailed ever since his time. The improvements of modern chemistry, by a more accurate analysis of these waters, have taught us the insufficiency of many of these opinions; but we remain as much as ever in doubt, as to the real causes. The prevalence of calculous complaints in some provinces of France, was a matter of inquiry among the learned societies in that kingdom; and at different times, imputed to the wine, the beer, and the water. A comparison between the different districts evinced the fallacy of all these conclusions. That calculous, as well as most other complaints, are hereditary, I shall offer one satisfactory proof. Mr. Ferreira, an ingenious surgeon from Sicily, did me the honour to attend a course of my lectures. After having attentively listened to my remarks on hereditary diseases, he asked me, Why I had not included calculus among them? This led to a conversation, during which, I discovered the great advantage he possessed, in tracing these hereditary properties in the human race, from the great caution with which the Sicilian nobility preserve the purity of their blood, by continual intermarriages, each in their own family. This is carried so far, that one family in which elephantiasis is hereditary, disdains an alliance with any other, on account of their own elevated rank.

"I cannot help suspecting, that it will hereafter be found, that gôitre and cretinism are endemial in certain districts, from no other cause than this hereditary property. M. Gautier, who has written a long account of gôitre, assures us, that it is not hereditary; yet, he afterwards adds, 'the first circumstance noticed, is a weak constitution derived from parents, which may contribute to give a predisposition to the disease.' A general opinion prevails, that gôitre is principally confined to mountainous situations; yet no one pretends to say, that every mountainous situation produces gôitre.

"Dr. Barton, Professor of Materia Medica, &c. in Pennsylvania, enumerates the various causes assigned for this disease. These are principally impregnation of the waters with calcareous and other matters, exposure to cold and drinking cold water, insects inhabiting the water, unwholesome food, and a residence in confined vallies. Dr. Barton easily shews the insufficiency of such causes, and concludes, with imputing the disease to those exhalations of marsh miasmata which induce the intermittent fever. But surely there is the same want of uniformity between cause and effect in this as in all the other instances. Though the professor has no view to any hereditary properties, yet he mentions a family, consisting of father, mother, and four or five children, all afflicted with the gôitre; and in his description of the places, in which the disease is found, seems to speak of it in one part as confined to the Indian families, and in another, in which it is wholly restricted, to the Canadians; meaning, I presume, the Colonists. In that part of Kent which borders on Sussex, and also in the adjoining parts of the latter county, the swelled throat is extremely common, and rarely confined to a single person in the same family. Whether there could be traced any alliance in these different families, it would be very difficult to ascertain, as the disease is principally confined to the females, who change their names on marriage, and also to the lower class, whose family connections are not so easily traced. I admit there are difficulties in the way, if, as is said by some, the inferior animals are sometimes affected; I have not, however, heard that such is the case in Derbyshire, or any other part of England. At all events, we must admit, that if the disease is excited by any local causes, the predisposition to such excitement is hereditary.

"I have never been able to learn, that ideotcy is associated with the swelled throat, any where but in the Alps; and some authors, who have given us an account of the diseases of those parts, assure us, that the union of the two is by no means universal: we cannot suppose that one cause should produce two such effects, and it is well ascertained, that ideotcy is hereditary in all its stages. Of this, Haller gives a striking instance from his own knowledge, 'Ex duabus patriciis sororibus ob divitias maritos nactis cum tamen fatuis essent proximæ, novimus in nobilissimas gentes nunc a seculo retro ejus morbi manasse semina ut etiam in quarta generatione quintave omnium posterorum aliqui fatui supersint.'*—May we not then impute its general prevalence in that secluded spot, to the accidental settlement of a family in which it was hereditary to produce ideots, and to the frequent intermarriages of their descendants, which was very likely to happen, where poverty and the wildness of the country would prevent migration from, or colonization among them? It is true, that no person who has made observation on the spot, as far as my reading has extended, has taken this view of the question; but most of them visited those places under previous impressions, and all of them speak of numbers in the same family afflicted with these diseases. I have attempted a correspondence with an eminent surgeon in that part of Italy, but the unsettled state of the continent has hitherto prevented me from receiving any information."

